# Gold Fillings

**Published:** 1903-12

**Authors:** H. Elmer Trostil


### GOLD FILLINGS.

BY H. ELMER TROSTIL, D.D.S.

MANY gold fillings of the present day last but a very short time. Why? First, because they are made of cohesive gold throughout, packed in such a careless manner that the gold is not perfectly adapted to the base and the walls of the cavity. Secondly, because deep retaining pits are used and the walls at the cervix thereby weakened. Why should this be done when the most gratifying results may be obtained by the use of cohesive gold throughout the cavity without pits or deep undercuts?

To accomplish this, prepare all cavities retentive in form, the same as for porcelain inlays, and flow Cavitine, Gilbert's Antiseptic Cavity Lining, or other such like preparation over the bottom of sensitive cavities, so as to protect the dentinal tubuli from the phosphoric acid in the cement.

This done and the gold annealed ready for manipulation, take a very small quantity of cement mixed to the consistency of putty and place it at the bottom of the cavity and over this a piece of gold large enough to cover the bottom of the cavity well, which should be pressed firmly to place with two pluggers. In a short time the cement will have hardened and will cling firmly to the walls of the cavity and to the gold, just as it does in retaining an inlay. In addition to this, in large cavities it prevents the gold from showing through the tooth-structure, which is ofttimes quite as unsightly as is the gold showing on the surface.

Where gold fillings are inserted in teeth that are conspicuous, or where they are exposed to mastication, they look and wear much better if they are finished with platinum and gold-foil No. 60. Of course, care must be taken to have the cavity filled to such an extent that the margins are covered before using the heavy foil, or there will be danger of fracturing the same.

